# Does the distance to the cancer center affect psycho-oncological care and emergency visits of patients with IDH wild-type gliomas? A retrospective study

**DOI:** 10.1093/nop/npad023

**Published:** 2023-04-27

**Authors:** Anna Fischl, Michael Gerken, Philipp Roos, Tareq Haedenkamp, Andrea Hillberg, Monika Klinkhammer-Schalke, Oliver Kölbl, Ralf Linker, Martin Proescholdt, Tobias Pukrop, Markus J Riemenschneider, Nils Ole Schmidt, Ingrid Schön, Martin Vogelhuber, Peter Hau, Elisabeth Bumes

**Affiliations:** Department of Neurology and Wilhelm Sander-NeuroOncology Unit, Regensburg University Hospital, Regensburg, Germany; Center for Quality Assurance and Health Services Research, University of Regensburg, Regensburg, Germany; Department of Neurology and Wilhelm Sander-NeuroOncology Unit, Regensburg University Hospital, Regensburg, Germany; Department of Neurology and Wilhelm Sander-NeuroOncology Unit, Regensburg University Hospital, Regensburg, Germany; Department of Internal Medicine III, Regensburg University Hospital, Regensburg, Germany; Center for Quality Assurance and Health Services Research, University of Regensburg, Regensburg, Germany; Department of Radiotherapy, Regensburg University Hospital, Regensburg, Germany; Department of Neurology and Wilhelm Sander-NeuroOncology Unit, Regensburg University Hospital, Regensburg, Germany; Department of Neurosurgery, Regensburg University Hospital, Regensburg, Germany; Department of Internal Medicine III, Regensburg University Hospital, Regensburg, Germany; Department of Neuropathology, Regensburg University Hospital, Regensburg, Germany; Department of Neurosurgery, Regensburg University Hospital, Regensburg, Germany; Department of Internal Medicine III, Regensburg University Hospital, Regensburg, Germany; Department of Internal Medicine III, Regensburg University Hospital, Regensburg, Germany; Department of Neurology and Wilhelm Sander-NeuroOncology Unit, Regensburg University Hospital, Regensburg, Germany; Department of Neurology and Wilhelm Sander-NeuroOncology Unit, Regensburg University Hospital, Regensburg, Germany

**Keywords:** brain tumor, emergency, glioblastoma, psycho-oncology, spatial distance

## Abstract

**Background:**

Malignant isocitrate dehydrogenase wild-type (IDHwt) gliomas impose a high symptomatic and psychological burden. Wide distances from patients’ homes to cancer centers may affect the delivery of psycho-oncological care. Here, we investigated, in a large brain tumor center with a rural outreach, the initiation of psycho-oncological care depending on spatial distance and impact of psycho-oncological care on emergency visits.

**Methods:**

Electronic patient charts, the regional tumor registry, and interviews with the primary care physicians were used to investigate clinical data, psycho-oncological care, and emergency unit visits. Interrelations with socio-demographic, clinical, and treatment aspects were investigated using univariable and multivariable binary logistic regression analysis and the Pearson’s Chi-square test.

**Results:**

Of 491, 229 adult patients of this retrospective cohort fulfilled the inclusion criteria for analysis. During the last three months of their lives, 48.9% received at least one psycho-oncological consultation, and 37.1% visited the emergency unit at least once. The distance from the cancer center did neither affect the initiation of psycho-oncological care nor the rate of emergency unit visits. Receiving psycho-oncological care did not correlate with the frequency of emergency unit visits in the last three months of life.

**Conclusion:**

We conclude that the distance of IDHwt glioma patients’ homes from their cancer center, even in a rural area, does not significantly influence the rate of psycho-oncological care.

Usually, malignant gliomas with isocitrate dehydrogenase wild-type (IDHwt) status are not curable.^1^ Patients frequently suffer from severe symptom burden^2^ and need a close medical/supportive network. Diagnosis and treatment are best organized within dedicated brain tumor cancer centers^3^ and psycho-oncological care is a pivotal element of these networks.^4^ Symptoms include focal neurological handicaps, epileptic seizures, brain edema,^5^ and cognitive deficits^6^ leading to restrictions on daily activity and health-related quality of life (HR-QoL).^7,8^ The extent of symptoms affects the need for psycho-oncological care and the rate of emergency presentations.^9,10^

As a result, a high-level medical and supportive network is a mainstay of a comprehensive treatment strategy. A central tool for improving HR-QoL is the early initiation of psycho-oncological care,^11^ which provides personalized concepts of care^12^ and is evaluated best with patient-reported outcome (PRO) measures.^13–15^ Psycho-oncology also fuels the supportive network by closely interacting with palliative care and hospice, social services, and supportive disciplines such as physiotherapy and sports therapy.^16^

The role of psycho-oncological care has been increasingly accounted for^17,18^ and has, eg, lead to integration of obligatory psycho-oncological assessments into neuro-oncology certification systems.^19^ In the German certification system for brain tumor centers, the respective effective care rate is reported with a median of about 22% of patients throughout all centers.^19^

Distance of patients’ homes from the cancer center may affect the initiation of psycho-oncological care^20^ and emergency treatment.^21^ Recent publications report that the rate of psycho-oncological care and HR-QoL^22^ in rural areas is worse compared to urban areas.^23^ It is also known that rural populations have a lower adherence to symptomatic and supportive treatment and a lower compliance.^24,25^ Data on the rate of emergency unit visits however remain diverse.^21,26^ However, no data have been reported so far in patients with IDHwt glioma.

In this retrospective study from a cancer center with a large academic primary brain tumor center, we hypothesized that patients with a longer distance to the cancer center had a lower rate of initiation of psycho-oncological care. We further hypothesized that the rate of emergency unit visits in the last 3 months before death was lower in patients who received psycho-oncological care. To test these hypotheses, we evaluated a cohort of 491 consecutively diagnosed patients with gliomas that were diagnosed between January 2014 and September 2020.

## Materials and Methods

### Patients’ Characteristics

**Figure 1.**
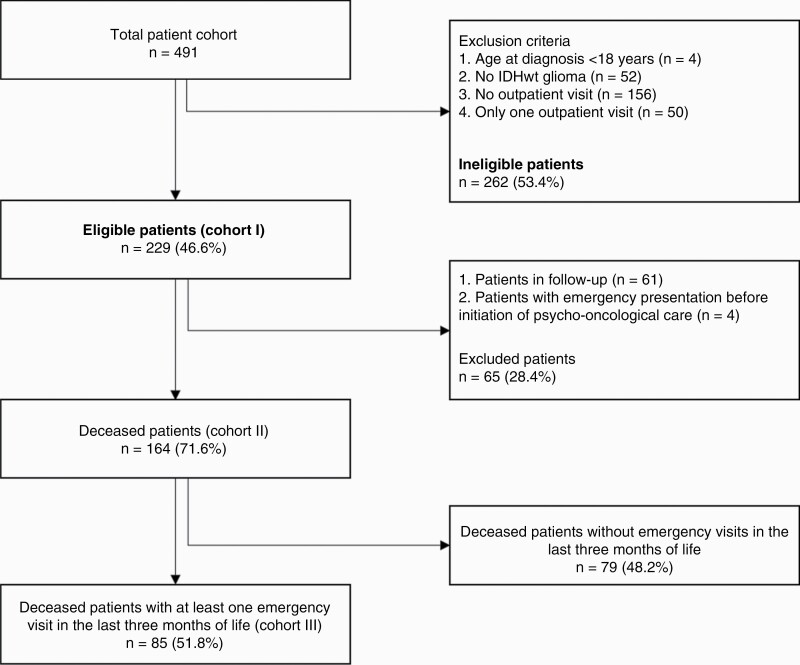
CONSORT diagram depicting patient numbers derived from inclusion and exclusion criteria and building of the groups. IDHwt, isocitrate dehydrogenase wild-type.

Patients with IDHwt glioma who were registered in the local tumor registry between January 2014 and September 2020 and filed in our hospital data management system were included in this study. Main inclusion criteria were neuropathological diagnosis of IDHwt glioma, age at diagnosis of 18 or above, and at least 2 outpatient visits after diagnosis (described in detail in Figure 1).

### Sociodemographic, Clinical, and Treatment Factors

Important demographic, neuropathological (according to WHO classification of 2016^27^), clinical, and treatment/supportive related aspects were drawn from the electronic medical records including the need for psycho-oncological care, measured with Hornheider Screening Instrument. Distance to cancer center was calculated as an airline from the patients’ homes to the cancer center. The rate of psycho-oncological care and emergency unit visits in the last 3 months before death were evaluated in interviews with primary care physicians and providers of psycho-oncological care.

### Distance to the Cancer Center

The postal codes of the patients´ adresses at time of the first diagnosis were drawn from the patients´ electronic medical records. Distances as airlines were estimated using the Google Maps distance measuring function and divided into 4 groups: group 1 with a radius of less than 30 km, group 2 with 30 to 59.9 km, group 3 with 60 to 89.9 km, and group 4 with 90 km or above.

### Psycho-Oncological Care

Initiation and offer of psycho-oncological care as well as quantity were documented by querying electronic patient charts and by a request to the psycho-oncologists. The need was evaluated with Hornheider Screening Instrument.^13^ Patients with a sum score of 4 and above and patients who requested psycho-oncological care received an initial consultation with a psycho-oncologist; the evaluation by Hornheider Screening Instrument was repeated during every visit, which routinely takes place every 2–4 months. If patients reached a sum score of 4 or above but declined consultation, it was recorded as “offer, no supply.”

During the initial psycho-oncological consultation, the offer of the psycho-oncological service was presented and a flyer with contact details was handed out. The patient’s individual issues and needs were addressed during psycho-oncological treatment. Aspects of the disease relevant to the patient were discussed and strategies for dealing with them were developed. The following topics were frequently addressed: Settling into the patient role, fears regarding illness or treatment, loss of control/helplessness, perception of one’s own feelings and needs, communication in the family, dealing with physical changes, dealing with death, and dying. Mainly, methods from cognitive behavioral therapy were applied, such as crisis intervention, resource activation, cognitive restructuring, psychoeducation, relaxation, and/or imaginative techniques.

Psycho-oncological care was provided at Regensburg University Hospital and District Hospital Regensburg. In no case, an additional external consultation was reported. Psycho-oncological care usually occurred during an inpatient stay or in combination with a planned consultation in the neuro-oncological outpatient clinic. If needed and desired, psycho-oncological consultations were performed independently of simultaneous medical outpatient visits. Only face-to-face appointments have been carried out and no additional telephone or video consultations took place.

### Statistics

Pseudonymized data were collected and recorded in charts in IBM SPSS (Chicago, IL, USA) Statistics (Version 25). Continuous data are expressed as means, medians, standard deviations, minima, and maxima. In the case of a normal distribution of the continuous variables, the Student’s *t-*test was performed to compare mean values. Mann–Whitney *U* test was used when this criterion was not met. Categorical variables were described as absolute frequencies and relative percentages. The independence between categorical variables was tested with Pearson’s Chi-square test. If the sample size was too small, Fisher’s exact test was applied. The level of significance was set at *P* < .05.

The correlation of clinical aspects, psycho-oncological care, and emergency visits was investigated with univariable and multivariable binary logistic regression analysis. The confounders for each analysis, which were selected on clinical experience, are listed in the legend of the respective table.

The cut-off *P*-value in the univariable binary logistic regression analyses was defined as *P* < .2 as a selection criterion for the multivariable analyses. If the investigated aspects met this criterion, they were included in the multivariable binary logistic regression analysis to reduce the bias of individual factors. The level of significance to test the influence of chosen aspects on receiving psycho-oncological care was set at *P* < .05.

### Ethical and Regulatory Framework

The study was approved by the Regensburg University Institutional Ethics Review Board (vote no. 19-1375-101). In accordance with local regulations, informed consent was not necessary due to the retrospective character of this analysis. The data protection concept of the Department of Neurology – NeuroOncology at Brain Tumor Center Regensburg which acts in the framework of the European General Data Protection Regulation and relevant national legislation was followed.

## Results

### Patients’ Cohorts

In total, 491 patients were identified by a data query at the Regional Cancer Registry. Of these, 229 (46.6%) fulfilled the predefined inclusion criteria of this retrospective study (cohort I, Figure 1). Until databank closure on September 30, 2020, 168 patients (73.4%) were deceased. Excluding 4 additional patients who had at least one emergency unit visit before the first psycho-oncological contact we formed a subgroup for analysis of emergency visits (cohort II). Cohort III includes all deceased patients with at least one emergency unit visit in the last 3 months before death (*n* = 85). If follow-up could not be directly performed, because patients were deceased until May 2020 (*n* = 162; 70.7%), primary care physicians were interviewed. One hundred and fifty-eight primary care physicians (97.5%) were able to be identified; information was obtained in 132 cases (83.5%). Complete information was received in 118 cases (89.4%), whereas in 14 cases (10.6%) only partial information was acquired.

### Patients’ Characteristics, Socio-demographic, Clinical, and Treatment Factors

In cohort I (*n* = 229), 48.9% of patients (*n* = 112) received psycho-oncological care. Sociodemographic factors, mean age, and age range at diagnosis were equally distributed with slightly more male than female patients in both groups (Supplementary Tables S1 and S2). Except for histological diagnosis (according to WHO classification of 2016^27^) no significant differences could be observed. Diagnosis of glioblastoma was more prevalent than anaplastic astrocytoma (*n* = 210 vs. *n* = 19; *P* = .04) and more frequent in patients with psycho-oncological care (Supplementary Table S3). Treatment characteristics did not differ between the groups except for distribution of chemotherapy as first-line treatment (*P* = .037; with psycho-oncological care 98.2% vs. without psycho-oncological care 92.3%) (Supplementary Table S4). The prevalence of palliative care was significantly different between patients without and with psycho-oncological care (32.5% vs. 48.2%; *P* = .019) (Supplementary Table S5).

### Offer of Psycho-oncological Care and Supply Rate

In cohort I 154 patients (67.2%) received an offer of psycho-oncological care. Of these 72.7% (*n* = 112/154) received at least one psycho-oncological consultation. Thirty-two of 112 patients (28.6%) had just one consultation, 57 patients (50.9%) had 2–5, and 23 patients (20.5%) had more than 5. The maximum was 46 consultations. In most patients (*n* = 90, 80.4%), the first psycho-oncological contact occurred during primary therapy, in 15 patients (13.4%) in the first relapse, and in 7 patients (6.3%) in the second or later relapse.

### Correlation Between Psycho-oncological Care and Distance to Cancer Center

We next investigated whether the distance between patients’ homes and the cancer center, measured as airline, influences receiving psycho-oncological care. Pearson’s Chi-square test showed no significance (*P* = .336) (Table 1).

**Table 1.** Psycho-oncological care depending on the distance from patients’ homes to the cancer center

		Psycho-oncological care								
		Not received		Offer, no supply		Offer and supply		Total		*X* ^2^
		*n*	(%)	*n*	(%)	*n*	(%)	*n*	(%)	*P*
Distance group^*^	0.0–29.9 km	19	25.3	7	16.7	31	27.6	57	24.9	.336
	30.0–59.9 km	15	20.0	14	33.3	29	25.9	58	25.3	
	60.0–89.9 km	20	26.7	11	26.2	34	30.4	65	28.4	
	≥90 km	21	28.0	10	23.8	18	16.1	49	21.4	
	Total	75	100.0	42	100.0	112	100.0	229	100.0	

Abbreviation: *X*^2^, Pearson’s Chi-square test.

^*^Distance equals as the airline from patients’ home to the cancer center in groups.

In an extended analysis, patients without psycho-oncological care were excluded, and the independence of distance to the cancer center and the frequency of psycho-oncological care was investigated in the remaining patients (*n* = 154). Frequency of psycho-oncological care was grouped into “only offer,” “only one visit,” “two to five visits,” and “more than five visits.” Pearson’s chi-square test showed a slight tendency toward fewer visits when distance was longer (*P* = .117) (Supplementary Table S6).

### Influence of Sociodemographic, Distance, Clinical and Treatment Aspects on Receiving Psycho-oncological Care

A binary logistic regression analysis was performed to assess factors influencing psycho-oncological care. The multivariable binary logistic regression analysis was used for confirmation and verified a documented need for psycho-oncological care as significant (*P* = .026; OR: 2.150; 95% CI: 1.096–4.216). Patients with an expressed need for psycho-oncological care were therefore 2.15 times more likely to receive psycho-oncological care compared to patients without an identified need. In addition, a trend was shown for glioblastoma vs. astrocytoma (*P* = .075; OR: 2.926; 95% CI: 0.898–9.538). All other aspects, including distance, showed no significant influence on receiving psycho-oncological care (Table 2).

**Table 2.** Univariable and multivariable binary logistic regression analysis of the influence of selected^*^ clinical aspects on receiving psycho-oncological care (*n* = 229)

Variable	Category (*n*)	Univariable binary logistic regression analysis				Multivariable binary logistic regression analysis^*^			
		*P*	OR	Lower 95%-CI	Upper 95%-CI	*P*	OR	Lower 95%-CI	Upper 95%-CI
Age groups at diagnosis	20.0–49.9 (30)		1.000				1.000		
	50.0–69.9 (160)	0.170	1.743	0.788	3.856	0.471	1.455	0.525	4.032
	70.0–9.9 (39)	0.727	0.840	0.315	2.239	0.445	0.607	0.168	2.187
Distance group^#^	0.0–29.9 km (57)		1.000				1.000		
	30.0–9.9 km (58)	0.638	0.839	0.403	1.745	0.467	1.403	0.563	3.498
	60.0–89.9 km (65)	0.818	0.920	0.451	1.877	0.339	1.529	0.640	3.657
	≥ 90 km (49)	0.071	0.487	0.223	1.063	0.314	0.624	0.249	1.564
Histological diagnosis	Anapl. astrocytoma (19)		1.000				1.000		
	Glioblastoma (210)	0.048	2.909	1.011	8.365	0.075	2.926	0.898	9.538
KPS group (at first visit)	<90 (89)		1.000				1.000		
	90–100 (135)	0.288	0.747	0.437	1.278	0.414	0.751	0.378	1.493
Depression (at first visit)	No (204)		1.000				1.000		
	Yes (25)	0.115	2.000	0.845	4.734	0.283	1.879	0.595	5.935
In need for POC^$^	No (< 4) (97)		1.000				1.000		
	Yes (≥ 4) (75)	0.092	1.687	0.919	3.100	0.026	2.150	1.096	4.216

Abbreviations: Anapl., anaplastic; CI, confidence interval; HSI, Hornheider Screening Instrument; OR, odds ratio; POC, psycho-oncological care.

^*^Selected clinical aspects (sex, age at diagnosis in years, distance from patients’ home to the cancer center, histological diagnosis, tumor localization, KPS at the first visit, symptomatic epilepsy at the first visit, depression at the first visit, need for psycho-oncological care, measured with Hornheider Screening Instrument, at the first outpatient visit) were tested for significance at level 0.2 and, if they met this criterion, were included in the multivariable binary logistic regression analysis for final assessment.

^#^Distance equals as the airline in groups from patients’ homes to the cancer center.

^$^Measured with Hornheider Screening Instrument at the first visit.

### Rate and Patterns of Emergency Unit Visits

The rate of emergency visits was investigated in cohort II (*n* = 164) including all patients deceased at the end of data acquisition with the exception of 4 patients who had at least 1 emergency unit visit before the first psycho-oncological consultation.

In total, 50.6% of patients (*n* = 83) in cohort II received psycho-oncological care. Demographic and clinical aspects in both groups were comparable (Supplementary Table S7). The proportion of patients without psycho-oncological care increased slightly, but not significantly, in correlation to the distance to the cancer center (*P* = .069) (Supplementary Table S7). Patients of cohort II with psycho-oncological care had more frequent surgery in relapse (*P* = .033) and were more likely to receive radiotherapy in relapse (*P* = .003) (Supplementary Table S8).

Deceased patients (54.2%) with psycho-oncological care had at least one emergency visit within the last 3 months of their life, compared to 49.4% of patients without psycho-oncological care (*n* = 81, 49.4%) (Supplementary Table S9). In total, 85 emergency unit visits were recorded. The mean number of all emergency unit visits was 0.94, with no significant differences between the groups (*P* = .564), and a maximum of 4 emergency unit visits.

Our analysis of whether distance from patients’ homes to the cancer center influenced the number of emergency unit visits showed a slight tendency for fewer visits with greater distance (*P* = .100) (Supplementary Table S10).

### Impact of Psycho-oncological Care on Emergency Visits

We next analyzed, if psycho-oncological care influences occurrence or reasons for emergency visits in the cohort of deceased patients (cohort II).

Here, we did not observe any influence of psycho-oncology care on the occurrence of emergency unit visits (Supplementary Table S11). We however did find a reduced risk of an emergency unit visit for older patients (>60 years) regardless of psycho-oncological care, who had a 0.247-fold risk compared to patients younger than 60 years (Supplementary Table S11). We further explored the influence of psycho-oncological care on the occurrence of selected clinical symptoms that commonly lead to emergency unit visits: signs of intracranial pressure, seizures, focal neurological symptoms due to tumor progression, infections, pain of other reasons, side effects of treatment and a remnant category that compiled several other reasons. Signs of intracranial pressure were significantly more prevalent in patients with psycho-oncological care (*P* = .019) (Supplementary Table S12).

Next, the influence of psycho-oncological care on reasons for presentation to the emergency unit was investigated. In our univariable and multivariable binary logistic regression analysis, no significant correlation with psycho-oncology care was observed (Supplementary Table S13).

For a sensitivity analysis, we investigated deceased patients, who presented at least once to the emergency unit in the last 3 months of life, in terms of the mean number of emergency visits (cohort III; *n* = 85) (Supplementary Tables S14 and S15). Here, 37 of 45 patients with psycho-oncological care were seen in the emergency unit in median 7.0 months after the last psycho-oncological consultation (minimum 0.5 months, maximum 23.3 months). In eight cases, emergency unit visits occurred during ongoing psycho-oncological treatment.

Multiple linear regression analysis verified a significant correlation (*P* = .007), with patients with psycho-oncological care having on average of 0.324 more emergency unit visits due to signs of intracranial pressure (*B*: 0.324; 95% CI: 0.070–0.413) (Supplementary Table S16).

## Discussion

In this retrospective study, we evaluated the impact of distance between patients’ homes and the cancer center on the initiation of psycho-oncological care and the rate and reasons for emergency unit visits in patients with IDHwt gliomas. Correlation of the occurrence of psycho-oncological care with rate and reasons for emergency visits, socio-demographic, clinical, and treatment aspects were investigated using univariable and multivariable binary logistic regression analysis.

Here, we prove that the distance between patients’ homes and the cancer center has no significant influence on the initiation and rate of psycho-oncological consultations, and emergency unit visits. A Hornheider Screening Instrument score ≥4 points was the only significant predictor for receiving psycho-oncological care, corroborating the validity of Hornheider Screening Instrument as a screening instrument.^13^

We found that a high rate of 48.9% of patients received at least one consultation at a psycho-oncologist. Literature on psycho-oncological care in patients with glioma is sparse, however, data are available in other tumor entities. In comparison, 49.8% of mixed cancer patients used this support,^28^ and 36.7% of patients with prostate cancer.^17^ An increased rate of psycho-oncological care has been frequently described for female patients in groups of mixed tumors,^29^ but this cannot be replicated in the field of brain tumors^6,30^ or in this study, which was not statistically powered to detect differences in gender distribution. Slightly half of the patients received 2–5 psycho-oncological consultations, while 28.6% were seen only once. Due to the retrospective nature of this analysis, it cannot be explained why psycho-oncological consultations were not always continued. However, according to clinical experience, major reasons may be refusal by patients, or organizational problems. Due to sparse data in the literature, it remains an open question why psycho-oncological care is not performed or not continued in patients with brain tumors despite a documented need. Further research on this point is warranted to improve the care of this highly distressed group of patients.

We observed the Hornheider Screening Instrument as a significant screening instrument for the need for psycho-oncological care in glioma patients. Fifty percent of the patients who received psycho-oncological care could be identified with Hornheider Screening Instrument, emphasizing the importance of PROs in identifying patients in need. This nicely correlates to literature, that 48.1% of lung cancer patients were identified with the Hornheider Screening Instrument.^14^ The proportion of patients in need of psycho-oncological care was lower (26.9%) in other publications with brain tumor patients, also using Hornheider Screening Instrument.^15^

Surprisingly, the distance between patients’ homes to the cancer center was not relevant for the initiation of psycho-oncological care. We hypothesize that factors other than distance play a greater role in the application of psycho-oncological care, however, we did not have the possibility to test this hypothesis within our retrospective dataset. At first glance, patients living in rural areas have more difficulties to access psycho-oncological care due to the lack of sufficient supply.^20,23^ In addition, counseling centers for psycho-oncological care are sought out more often in urban areas.^18^ On the other hand, living in a rural area can deteriorate mental health,^22^ which would highly mandate sufficient supply.

A cohort of 164 patients was selected to assess more accurately the rate of psycho-oncological care and emergency unit visits within the last 3 months of life. In total, 51.8% of these patients did present at least once to the emergency unit.

In literature, both results are reported, reduction of emergency unit visits in tumor patients in a rural environment^21^ and, on the opposite, an increase, eg, in colorectal carcinoma.^26^ In this first investigation in a brain tumor cohort, we show that the rate of emergency unit visits was independent of the distance between patients’ homes to the cancer center. Therefore, it can be assumed that emergency unit visits in patients with IDHwt glioma are executed, if necessary, regardless of the distance to the cancer center.

In our cohort of 164 patients, we found no significant correlation between psycho-oncological care and occurrence of emergency unit visits. However, this is not surprising, as many other factors are influencing the rate of emergency unit visits, such as supply of palliative care, pre-existing and concomitant diseases, and the home care situation. The influence of palliative care on emergency presentations in patients with mixed tumors is also known,^31^ but was not the main subject of this study. In conclusion, the impact of psycho-oncological care on the occurrence of emergency unit visits may be moderate at best because of many competing factors. In particular, the effect of palliative care in this context should be given more attention in future research.

Surprisingly, a significant positive correlation between psycho-oncological care and “signs of intracranial pressure” as a clinical symptom for emergency unit visits was observed and confirmed in the sensitivity analysis. Here, we can only speculate whether patients with a high tumor burden suffering from more severe clinical symptoms may receive more frequent psycho-oncological care. In turn, high tumor burden and thus increased risk of brain edema could cause more emergency presentations due to “signs of intracranial pressure.” Neither literature nor data from this cohort can sufficiently support this hypothesis.

Our study suffers from some weaknesses. First, this is a single-center study in a primary brain tumor center with a wide rural outreach. Results may differ in multi-center analysis or in centers with a different socio-demographic background or more urban population. However, the Regensburg Brain Tumor Center reflects very well the average quality parameters of the German certification system, which also include rates, eg, of psycho-oncological care.^19^ In addition, we were not able to generate a complete follow-up for psycho-oncological care in this already deceased population. However, this is notorious in similar studies.^28,29^

The study also has important advantages. We investigated a comparably high number of homogenous patients who were treated according to a strict pathway within one high-volume dedicated academic brain tumor center. In addition, this is the first study that investigated the influence of receiving psycho-oncological care on emergency unit visits in IDHwt glioma patients, generating important novel results that can build hypotheses for future research.

In summary, we found that the distance of IDHwt glioma patients´ homes to the cancer center does not significantly influence the rate of psycho-oncological care or emergency unit visits. We conclude that utmost efforts should be imposed on the maximal integration of supportive psycho-oncological care on a daily-practice basis. The kind and frequency of interventions should be optimized to relieve the symptomatic and psycho-social burden. Further, influencing factors on emergency unit visits should be identified to develop measures that could decrease the frequency of emergency presentations and improve care in this highly distressed group of patients with IDHwt glioma.

## Supplementary Material

npad023_suppl_Supplementary_Material
