# Supplementary material for: Does the distance to the cancer center affect psycho-oncological care and emergency visits of patients with IDH wild-type gliomas? A retrospective study
Source: Neurooncol Pract. 2023 Apr 27;10(5):446–53. doi: 10.1093/nop/npad023 (PMC10502780; doi:10.1093/nop/npad023)
Supplement: npad023_suppl_Supplementary_Material [file npad023_suppl_supplementary_material.docx]

**Suppl. Table S1** Sociodemographic characteristics in patients without and with psycho-oncological care in cohort I (n = 229).

|  | | Psycho-oncological care | | | | | |  |
| --- | --- | --- | --- | --- | --- | --- | --- | --- |
|  |  | No | | Yes | | Total | | X^2^ |
|  |  | n | (%) | n | (%) | n | (%) | *p* |
| Sex | Male | 63 | 53.8% | 65 | 58.0% | 128 | 55.9% | 0.523 |
|  | Female | 54 | 46.2% | 47 | 42.0% | 101 | 44.1% |  |
|  | Total | 117 | 100.0% | 112 | 100.0% | 229 | 100.0% |  |
| Age group at diagnosis | 20.0 – 49.9 | 18 | 15.4% | 12 | 10.7% | 30 | 13.1% |  |
|  | 50.0 – 69.9 | 74 | 63.2% | 86 | 76.8% | 160 | 69.9% | 0.078 |
|  | 70.0 – 99.9 | 25 | 21.4% | 14 | 12.5% | 39 | 17.0% |  |
|  | Total | 117 | 100.0% | 112 | 100.0% | 229 | 100.0% |  |
| Distance from patients’ home to cancer center | 0.0 – 29.9 km | 26 | 22.2% | 31 | 27.7% | 57 | 24.9% |  |
|  | 30.0 – 59.9 km | 29 | 24.8% | 29 | 25.9% | 58 | 25.3% |  |
|  | 60.0 – 89.9 km | 31 | 26.5% | 34 | 30.4% | 65 | 28.4% | 0.270 |
|  | ≥ 90 km | 31 | 26.5% | 18 | 16.1% | 49 | 21.4% |  |
|  | Total | 117 | 100.0% | 112 | 100.0% | 229 | 100.0% |  |

**Abbreviation:** n, valid number; X^2^, Pearson’s Chi-square test.

**Suppl. Table S2** Mean, median, minimum, maximum, range, standard deviation of age at diagnosis in patients without and with psycho-oncological care in cohort I (n = 229).

|  | | Psycho-oncological care | | | t-Test |
| --- | --- | --- | --- | --- | --- |
|  |  | No | Yes | Total | *p* |
| Age at diagnosis | Valid number | 117 | 112 | 229 | 0.536 |
|  | Mean | 60.4 | 59.5 | 60.0 |  |
|  | Median | 60.4 | 60.8 | 60.8 |  |
|  | Minimum | 21.4 | 22.6 | 21.4 |  |
|  | Maximum | 83.8 | 84.0 | 84.0 |  |
|  | Range | 62.4 | 61.4 | 62.7 |  |
|  | Standard deviation | 11.4 | 10.6 | 11.0 |  |

**Suppl. Table S3** Clinical characteristics of patients without and with psycho-oncological care in cohort I (n = 229).

|  | | Psycho-oncological care | | | | | |  |
| --- | --- | --- | --- | --- | --- | --- | --- | --- |
|  |  | No | | Yes | | Total | | X^2^ |
|  |  | n | (%) | n | (%) | n | (%) | *p* |
| Histological diagnosis | Anaplastic astrocytoma, IDHwt  (WHO III) | 14 | 12.0% | 5 | 4.5% | 19 | 8.3% | 0.040 |
|  | Glioblastoma, IDHwt (WHO IV) | 103 | 88.0% | 107 | 95.5% | 210 | 91.7% |  |
|  | Total | 117 | 100.0% | 112 | 100.0% | 229 | 100.0% |  |
| Tumor localization (ICD-10) | C71.0 Cerebrum | 0 | 0.0% | 3 | 2.7% | 3 | 1.3% | 0.564 |
|  | C71.1 Frontal lobe | 29 | 24.8% | 22 | 19.6% | 51 | 22.3% |  |
|  | C71.2 Temporal lobe | 35 | 29.9% | 31 | 27.7% | 66 | 28.8% |  |
|  | C71.3 Parietal lobe | 18 | 15.4% | 17 | 15.2% | 35 | 15.3% |  |
|  | C71.4 Occipital lobe | 5 | 4.3% | 7 | 6.3% | 12 | 5.2% |  |
|  | C71.6 Cerebellum | 0 | 0.0% | 1 | 0.9% | 1 | 0.4% |  |
|  | C71.7 Brain stem | 1 | 0.9% | 0 | 0.0% | 1 | 0.4% |  |
|  | C71.8 Brain, subareas overlapping | 17 | 14.5% | 22 | 19.6% | 39 | 17.0% |  |
|  | C71.9 Brain, not specified | 11 | 9.4% | 9 | 8.0% | 20 | 8.7% |  |
|  | C72.0 Spinal chord | 1 | 0.9% | 0 | 0.0% | 1 | 0.4% |  |
|  | Total | 117 | 100.0% | 112 | 100.0% | 229 | 100.0% |  |
| MGMT promoter methylation status | No methylation | 55 | 47.0% | 60 | 53.6% | 115 | 50.2% | 0.484 |
|  | Methylation | 58 | 49.6% | 50 | 44.6% | 108 | 47.2% |  |
|  | Not specified | 4 | 3.4% | 2 | 1.8% | 6 | 2.6% |  |
|  | Total | 117 | 100.0% | 112 | 100.0% | 229 | 100.0% |  |
| KPS group  (first visit) | < 90 | 41 | 36.3% | 48 | 43.2% | 89 | 39.7% | 0.287 |
|  | 90 – 100 | 72 | 63.7% | 63 | 56.8% | 135 | 60.3% |  |
|  | Total | 113 | 100.0% | 111 | 100.0% | 224 | 100.0% |  |
| Depression  (first visit) | No | 108 | 92.3% | 96 | 85.7% | 204 | 89.1% | 0.110 |
|  | Yes | 9 | 7.7% | 16 | 14.3% | 25 | 10.9% |  |
|  | Total | 117 | 100.0% | 112 | 100.0% | 229 | 100.0% |  |
| Symptomatic epilepsy  (first visit) | No | 49 | 41.9% | 54 | 48.2% | 103 | 45.0% | 0.335 |
|  | Yes | 68 | 58.1% | 58 | 51.8% | 126 | 55.0% |  |
|  | Total | 117 | 100.0% | 112 | 100.0% | 229 | 100.0% |  |

**Abbreviation:** ICD-10, International Statistical Classification of Diseases and Related Health Problems- Revision 10; MGMT, O6-Methylguanin-DNS-Methyltransferase; n, valid number; X^2^, Pearson’s Chi-square test.

**Suppl. Table S4** Treatment characteristics in patients without and with psycho-oncological care in cohort I (n = 229).

|  | | Psycho-oncological care | | | | | |  |
| --- | --- | --- | --- | --- | --- | --- | --- | --- |
|  |  | No | | Yes | | Total | | X^2^ |
|  |  | n | (%) | n | (%) | n | (%) | *p* |
| First-line treatment: surgery | Biopsy | 20 | 17.1% | 18 | 16.1% | 38 | 16.6% | 0.545 |
|  | Incomplete resection | 25 | 21.4% | 33 | 29.5% | 58 | 25.3% |  |
|  | Macroscopic complete resection | 61 | 52.1% | 53 | 47.3% | 114 | 49.8% |  |
|  | Not specified | 11 | 9.4% | 8 | 7.1% | 19 | 8.3% |  |
|  | Total | 117 | 100.0% | 112 | 100.0% | 229 | 100.0% |  |
| First-line treatment:  radiotherapy | No | 10 | 8.5% | 5 | 4.5% | 15 | 6.6% | 0.212 |
|  | Yes | 107 | 91.5% | 107 | 95.5% | 214 | 93.4% |  |
|  | Total | 117 | 100.0% | 112 | 100.0% | 229 | 100.0% |  |
| First-line treatment:  chemotherapy | No | 9 | 7.7% | 2 | 1.8% | 11 | 4.8% | 0.037 |
|  | Yes | 108 | 92.3% | 110 | 98.2% | 218 | 95.2% |  |
|  | Total | 117 | 100.0% | 112 | 100.0% | 229 | 100.0% |  |
| First-line treatment: tumor treating-fields | No | 86 | 73.5% | 75 | 67.0% | 161 | 70.3% | 0.279 |
|  | Yes | 31 | 26.5% | 37 | 33.0% | 68 | 29.7% |  |
|  | Total | 117 | 100.0% | 112 | 100.0% | 229 | 100.0% |  |

**Abbreviation:** n, valid number; X^2^, Pearson’s Chi-square test.

**Suppl. Table S5** Need for psycho-oncological care and occurrence of palliative care in patients without and with psycho-oncological care in cohort I (n = 229).

|  | | Psycho-oncological care | | | | | |  |
| --- | --- | --- | --- | --- | --- | --- | --- | --- |
|  |  | No | | Yes | | Total | | X^2^ |
|  |  | n | (%) | n | (%) | n | (%) | *p* |
| In need for POC^*^  (first visit) | No (< 4) | 54 | 62.8% | 43 | 50.0% | 97 | 56.4% | 0.091 |
|  | Yes (≥ 4) | 32 | 37.2% | 43 | 50.0% | 75 | 43.6% |  |
|  | Total | 86 | 100.0% | 86 | 100.0% | 172 | 100.0% |  |
| Palliative care | No | 62 | 53.0% | 51 | 45.5% | 113 | 49.3% | 0.019 |
|  | Yes | 38 | 32.5% | 54 | 48.2% | 92 | 40.2% |  |
|  | No data available | 17 | 14.5% | 7 | 6.3% | 24 | 10.5% |  |
|  | Total | 117 | 100.0% | 112 | 100.0% | 229 | 100.0% |  |

**Abbreviation:** POC, Psycho-oncological care; n, valid number; X^2^, Pearson’s Chi-square test.

^*^Measured with Hornheider Screening Instrument.

**Suppl. Table S6** Frequency of psycho-oncological consultations depends on the distance from patients’ homes to the cancer center. Patients without psycho-oncological care are not included.

|  | | | Quantity of psycho-oncological care meetings | | | | | X^2^ |
| --- | --- | --- | --- | --- | --- | --- | --- | --- |
|  |  |  | Only offer | Only one^#^ | ≥ 2^#^ | ≥ 6^#^ | Total | *p* |
| Distance from patients’ home  to cancer center | 1. – 29.9 km | n^*^ | 7 (16.7%) | 8 (25.0%) | 14 (24.6%) | 9 (39.1%) | 38 (24.7%) | 0.117 |
|  | 30.0 – 59.9 km | n^*^ | 14 (33.3%) | 4 (12.5%) | 20 (35.1%) | 5 (21.7%) | 43 (27.9%) |  |
|  | 60.0 – 89.9 km | n^*^ | 11 (26.2%) | 14 (43.8%) | 14 (24.6%) | 6 (26.2%) | 45 (29.2%) |  |
|  | ≥ 90 km | n^*^ | 10 (23.8%) | 6 (18.8%) | 9 (15.7%) | 3 (13.0%) | 28 (18.2%) |  |
|  | Total | n^*^ | 42 (100.0%) | 32 (100.0%) | 57 (100.0%) | 23 (100.0%) | 154 (100.0%) |  |

**Abbreviation:** X^2^, Pearson’s Chi-square test.

n^*^: valid number.

^#^Numbers quantify the rate of meetings.

**Suppl. Table S7** Demographic and clinical aspects of patients without and with psycho-oncological care in cohort II (n = 164).

|  | | Psycho-oncological care | | | | | |  |
| --- | --- | --- | --- | --- | --- | --- | --- | --- |
|  |  | No | | Yes | | Total | | X^2^ |
|  |  | n | (%) | n | (%) | n | (%) | *p* |
| Sex | Male | 44 | 54.3% | 49 | 59.0% | 93 | 56.7% | 0.542 |
|  | Female | 37 | 45.7% | 34 | 41.0% | 71 | 43.3% |  |
|  | Total | 81 | 100.0% | 83 | 100.0% | 164 | 100.0% |  |
| Age group at diagnosis | 20.0 – 59.9 | 39 | 48.1% | 37 | 44.6% | 76 | 46.3% | 0.647 |
|  | 60.0 – 99.9 | 42 | 51.9% | 46 | 55.4% | 88 | 53.7% |  |
|  | Total | 81 | 100.0% | 83 | 100.0% | 164 | 100.0% |  |
| Distance from patient’s home to cancer center | 0.0 – 29.9 km | 14 | 17.3% | 25 | 30.1% | 39 | 23.8% | 0.069 |
|  | 30.0 – 59.9 km | 21 | 25.9% | 18 | 21.7% | 39 | 23.8% |  |
|  | 60.0 – 89.9 km | 22 | 27.2% | 27 | 32.5% | 49 | 29.9% |  |
|  | ≥ 90 km | 24 | 29.6% | 13 | 15.7% | 37 | 22.6% |  |
|  | Total | 81 | 100.0% | 83 | 100.0% | 164 | 100.0% |  |
| Histological diagnosis | Anaplastic astrocytoma, IDHwt (WHO III) | 10 | 12.3% | 4 | 4.8% | 14 | 8.5% | 0.085 |
|  | Glioblastoma, IDHwt (WHO IV) | 71 | 87.7% | 79 | 95.2% | 150 | 91.5% |  |
|  | Total | 81 | 100.0% | 83 | 100.0% | 164 | 100.0% |  |
| KPS group (90 days before death) | < 70 | 17 | 21.0% | 21 | 25.3% | 38 | 23.2% | 0.337 |
|  | 70 – 80 | 29 | 35.8% | 23 | 27.7% | 52 | 31.7% |  |
|  | 90 – 100 | 3 | 3.7% | 8 | 9.6% | 11 | 6.7% |  |
|  | Not specified | 32 | 39.5% | 31 | 37.3% | 63 | 38.4% |  |
|  | Total | 81 | 100.0% | 83 | 100.0% | 164 | 100.0% |  |

**Abbreviation:** n, valid number; X^2^, Pearson’s Chi-square test; IDHwt, Isocitrate dehydrogenase-wildtype.

**Suppl. Table S8** Pre-existing and disease-related patterns of patients without and with psycho-oncological care in cohort II (n = 164).

|  | | Psycho-oncological care | | | | | |  |
| --- | --- | --- | --- | --- | --- | --- | --- | --- |
|  |  | No | | Yes | | Total | | X^2^ |
|  |  | n | (%) | n | (%) | n | (%) | *p* |
| Symptomatic epilepsy  (last visit) | No | 22 | 27.2% | 28 | 33.7% | 50 | 30.5% | 0.361 |
|  | Yes | 59 | 72.8% | 55 | 66.3% | 114 | 69.5% |  |
|  | Total | 81 | 100.0% | 83 | 100.0% | 164 | 100.0% |  |
| Depression  (last visit) | No | 70 | 86.4% | 68 | 81.9% | 138 | 84.1% | 0.431 |
|  | Yes | 11 | 13.6% | 15 | 18.1% | 26 | 15.9% |  |
|  | Total | 81 | 100.0% | 83 | 100.0% | 164 | 100.0% |  |
| Levetiracetam intake  (last visit) | No | 37 | 45.7% | 39 | 47.0% | 76 | 46.3% | 0.867 |
|  | Yes | 44 | 54.3% | 44 | 53.0% | 88 | 53.7% |  |
|  | Total | 81 | 100.0% | 83 | 100.0% | 164 | 100.0% |  |
| Palliative care | No | 30 | 37.0% | 30 | 36.1% | 60 | 36.6% | 0.071 |
|  | Yes | 36 | 44.4% | 47 | 56.6% | 83 | 50.6% |  |
|  | No data available | 15 | 18.5% | 6 | 7.2% | 21 | 12.8% |  |
|  | Total | 81 | 100.0% | 83 | 100.0% | 164 | 100.0% |  |
| In Need of POC^*^  (last visit) | No (< 4) | 25 | 49.0% | 23 | 39.0% | 48 | 43.6% | 0.290 |
|  | Yes (≥ 4) | 26 | 51.0% | 36 | 61.0% | 62 | 56.4% |  |
|  | Total | 51 | 100.0% | 59 | 100.0% | 110 | 100.0% |  |
| Surgery at tumor progression | 1 | 21 | 87.5% | 18 | 72.0% | 39 | 79.6% | 0.033 |
|  | 2 | 3 | 12.5% | 7 | 28.0% | 10 | 20.4% |  |
|  | Total | 24 | 100.0% | 25 | 100.0% | 49 | 100.0% |  |
| Radiotherapy at tumor progression | 1 | 16 | 84.2% | 31 | 88.6% | 47 | 87.0% | 0.003 |
|  | 2 | 3 | 15.8% | 4 | 11.4% | 7 | 13.0% |  |
|  | Total | 19 | 100.0% | 35 | 100.0% | 54 | 100.0% |  |
| Chemotherapy at tumor progression | 1 | 17 | 43.6% | 23 | 43.4% | 40 | 43.5% | 0.985 |
|  | 2 | 22 | 56.4% | 30 | 56.6% | 52 | 56.5% |  |
|  | Total | 39 | 100.0% | 53 | 100.0% | 92 | 100.0% |  |

**Abbreviation:** POC, Psycho-oncological care; n, valid number; X^2^, Pearson’s Chi-square test.

^*^Measured with Hornheider Screening Instrument.

**Suppl. Table S9** Number of emergency presentations of patients without and with psycho-oncological care in cohort II (n = 164).

|  | | Psycho-oncological care | | | | | |  |
| --- | --- | --- | --- | --- | --- | --- | --- | --- |
|  |  | No | | Yes | | Total | | T-Test |
|  |  | n | (%) | n | (%) | n | (%) | *p* |
| Number of emergency presentations | 0 | 41 | 50.6% | 38 | 45.8% | 79 | 48.2% | 0.537 |
|  | 1 | 16 | 19.8% | 24 | 28.9% | 40 | 24.4% |  |
|  | 2 | 17 | 21.0% | 11 | 13.3% | 28 | 17.1% |  |
|  | 3 | 3 | 3.7% | 7 | 8.4% | 10 | 6.1% |  |
|  | 4 | 4 | 4.9% | 3 | 3.6% | 7 | 4.3% |  |
|  | Total | 81 | 100.0% | 83 | 100.0% | 164 | 100.0% |  |

**Abbreviation:** n: valid number.

**Suppl. Table S10** Number of emergency presentations depending on the distance from patients’ homes to the cancer center in cohort II (n = 164).

|  | | | Number of emergency presentations | | | | X^2^ |
| --- | --- | --- | --- | --- | --- | --- | --- |
|  |  |  | No emergency | One emergency | >1 emergency | Total | *p* |
| Distance from patient’s home to cancer center | 0.0 – 29.9 km | n^*^ | 8 (14.3%) | 7 (17.5%) | 24 (35.3%) | 39 (23.8%) | 0.100 |
|  | 30.0 – 59.9 km | n^*^ | 13 (23.2%) | 11 (27.5%) | 15 (22.1%) | 39 (23.8%) |  |
|  | 60.0 – 89.9 km | n^*^ | 22 (39.3%) | 13 (32.5%) | 14 (20.6%) | 49 (29.9%) |  |
|  | ≥ 90 km | n^*^ | 13 (23.2%) | 9 (22.5%) | 15 (22.1%) | 37 (22.6%) |  |
|  | Total | n^*^ | 56 (100.0%) | 40 (100.0%) | 68 (100.0%) | 164 (100.0%) |  |

**Abbreviation:** X^2^, Pearson’s Chi-square test.

n^*^: valid number.

**Suppl. Table S11** Univariable and multivariable binary logistic regression analysis of the influence of psycho-oncological care and clinical aspects^*^ on the occurrence of an emergency presentation in the last three months before death in cohort II (n = 164).

| Variable | | Category (n) | Univariable binary logistic  regression analysis | | | | Multivariable binary logistic regression analysis^*^ | | | |
| --- | --- | --- | --- | --- | --- | --- | --- | --- | --- | --- |
|  |  |  | *p* | OR | Lower 95%-CI | Upper 95%-CI | *p* | OR | Lower  95%-CI | Upper 95%-CI |
| Psycho-oncological care | No (81) | |  | 1.000 |  |  |  | 1.000 |  |  |
|  | Yes (83) | | 0.536 | 1.214 | 0.657 | 2.241 | 0.666 | 0.814 | 0.319 | 2.076 |
| Age at diagnosis | 20.0 – 59.9 (76) | |  | 1.000 |  |  |  | 1.000 |  |  |
|  | 60.0 – 99.9 (88) | | 0.149 | 0.634 | 0.342 | 1.178 | 0.007 | 0.247 | 0.089 | 0.682 |
| KPS (three months before death) | Increase: 10% | | 0.092 | 1.022 | 0.996 | 1.047 | 0.083 | 1.026 | 0.997 | 1.057 |
| Number of resection  (for progression) | Increase: 1 surgery in progression | | 0.031 | 1.783 | 1.055 | 3.016 | 0.277 | 1.671 | 0.662 | 4.217 |
| Palliative care | No (60) | |  | 1.000 |  |  |  | 1.000 |  |  |
|  | Yes (83) | | 0.696 | 1.142 | 0.586 | 2.227 | 0.175 | 2.003 | 0.735 | 5.459 |
|  | No data available^#^ (21) | |  |  |  |  |  |  |  |  |

**Abbreviation:** OR, Odds Ratio; CI, Confidence Interval**.**

^*^Selected clinical aspects (sex, age at diagnosis in years, histological diagnosis, KPS six months before death, the total number of surgeries, radiotherapies, chemotherapies for progression therapy, symptomatic epilepsy at the last visit, depression at the last visit, palliative care, need for psycho-oncological care, measured with Hornheider Screening Instrument, at the last visit) were tested for significance at level 0.2 and, if they met the criterion, were included in the multivariable binary logistic regression analysis for adjustment.

^#^Results not shown.

**Suppl. Table S12** Number and proportion of common reasons for emergency unit presentations of patients without and with psycho-oncological care in cohort II (n = 164).

|  | | Psycho-oncological care | | | | | |  |
| --- | --- | --- | --- | --- | --- | --- | --- | --- |
|  |  | No | | Yes | | Total | | X^2^ |
|  |  | n | (%) | n | (%) | n | (%) | *p* |
| Signs of intracranial pressure | No | 79 | 97.5% | 73 | 88.0% | 152 | 92.7% | 0.019 |
|  | Yes | 2 | 2.5% | 10 | 12.0% | 12 | 7.3% |  |
|  | Total | 81 | 100.0% | 83 | 100.0% | 164 | 100.0% |  |
| Seizures | No | 69 | 85.2% | 76 | 91.6% | 145 | 88.4% | 0.202 |
|  | Yes | 12 | 14.8% | 7 | 8.4% | 19 | 11.6% |  |
|  | Total | 81 | 100.0% | 83 | 100.0% | 164 | 100.0% |  |
| Symptoms due to progression | No | 66 | 81.5% | 68 | 81.9% | 134 | 81.7% | 0.941 |
|  | Yes | 15 | 18.5% | 15 | 18.1% | 30 | 18.3% |  |
|  | Total | 81 | 100.0% | 83 | 100.0% | 164 | 100.0% |  |
| Infection | No | 74 | 91.4% | 71 | 85.5% | 145 | 88.4% | 0.245 |
|  | Yes | 7 | 8.6% | 12 | 14.5% | 19 | 11.6% |  |
|  | Total | 81 | 100.0% | 83 | 100.0% | 164 | 100.0% |  |
| Pain | No | 77 | 95.1% | 77 | 92.8% | 154 | 93.9% | 0.636 |
|  | Yes | 4 | 4.9% | 6 | 7.2% | 10 | 6.1% |  |
|  | Total | 81 | 100.0% | 83 | 100.0% | 164 | 100.0% |  |
| Treatment side effects | No | 75 | 92.6% | 75 | 90.4% | 150 | 91.5% | 0.609 |
|  | Yes | 6 | 7.4% | 8 | 9.6% | 14 | 8.5% |  |
|  | Total | 81 | 100.0% | 83 | 100.0% | 164 | 100.0% |  |
| Other | No | 67 | 82.7% | 72 | 86.7% | 139 | 84.8% | 0.473 |
|  | Yes | 14 | 17.3% | 11 | 13.3% | 25 | 15.2% |  |
|  | Total | 81 | 100.0% | 83 | 100.0% | 164 | 100.0% |  |

**Abbreviation:** n, valid number; X^2^, Pearson’s Chi-square test.

**Suppl. Table S13** Univariable and multivariable binary logistic regression analysis for the influence of psycho-oncological care on the occurrence of emergency presentations for specific reasons in the last three months before death in cohort II (n = 164).

| Reason for emergency presentation | Univariable binary logistic  regression analysis | | | | | | | Multivariable binary logistic regression analysis^*^ | | | | | | | |
| --- | --- | --- | --- | --- | --- | --- | --- | --- | --- | --- | --- | --- | --- | --- | --- |
|  | *p* | | OR | Lower 95%-CI | | Upper 95%-CI | | *p* | OR | | Lower 95%-CI | | Upper 95%-CI | | |
| Signs of intracranial pressure | | 0.033 | 5.411 | | 1.147 | 25.523 | 0.052 | | | 5.115 | | 0.984 | | 26.580 |  |
| Seizures | | 0.207 | 0.530 | | 0.197 | 1.422 | 0.287 | | | 0.450 | | 0.103 | | 1.958 |  |
| Symptoms due to tumor progression | | 0.941 | 0.971 | | 0.440 | 2.142 | 0.354 | | | 0.624 | | 0.230 | | 1.691 |  |
| Infections | | 0.249 | 1.787 | | 0.666 | 4.796 | 0.245 | | | 1.803 | | 0.667 | | 4.870 |  |
| Pain | | 0.542 | 1.500 | | 0.407 | 5.526 | 0.643 | | | 1.385 | | 0.350 | | 5.470 |  |
| Treatment side effects | | 0.610 | 1.333 | | 0.441 | 4.029 | 0.119 | | | 8.124 | | 0.582 | | 113.444 |  |
| Other | | 0.474 | 0.731 | | 0.310 | 1.723 | 0.958 | | | 0.989 | | 0.301 | | 3.121 |  |

**Abbreviations:** OR, Odds Ratio; CI, Confidence Interval; POC, Psycho-oncological care; HSI, Hornheider Screening Instrument.

^*^Selected clinical aspects (sex, age at diagnosis in years, histological diagnosis, KPS six months before death, the total number of surgeries, radiotherapies, chemotherapies for progression therapy, symptomatic epilepsy, levetiracetam at the last visit, depression at the last visit, palliative care, need for psycho-oncological care, measured with Hornheider Screening Instrument, at the last visit) were tested for significance at level 0.2 and, if they met the criterion, were included in the multivariable binary logistic regression analysis for adjustment.

**Suppl. Table S14** Demographic and clinical data of patients without and with psycho-oncological care in cohort III (n = 85).

|  | | Psycho-oncological care | | | | | |  |
| --- | --- | --- | --- | --- | --- | --- | --- | --- |
|  |  | No | | Yes | | Total | | X^2^ |
|  |  | n | (%) | n | (%) | n | (%) | *p* |
| Sex | Male | 22 | 55.0% | 30 | 66.7% | 52 | 61.2% | 0.271 |
|  | Female | 18 | 45.0% | 15 | 33.3% | 33 | 38.8% |  |
|  | Total | 40 | 100.0% | 45 | 100.0% | 85 | 100.0% |  |
| Age group at diagnosis | 20.0 – 59.9 | 22 | 55.0% | 22 | 48.9% | 44 | 51.8% | 0.574 |
|  | 60.0 – 99.9 | 18 | 45.0% | 23 | 51.1% | 41 | 48.2% |  |
|  | Total | 40 | 100.0% | 45 | 100.0% | 85 | 100.0% |  |
| Histological diagnosis | Anaplastic astrocytoma, IDHwt (WHO III) | 5 | 12.5% | 1 | 2.2% | 6 | 7.1% | 0.095 |
|  | Glioblastoma, IDHwt (WHO IV) | 35 | 87.5% | 44 | 97.8% | 79 | 92.9% |  |
|  | Total | 40 | 100.0% | 45 | 100.0% | 85 | 100.0% |  |
| KPS group  (90 days before death) | < 70 | 10 | 25.0% | 9 | 20.0% | 19 | 22.4% | 0.284 |
|  | 70 – 80 | 20 | 50.0% | 18 | 40.0% | 38 | 44.7% |  |
|  | 90 – 100 | 2 | 5.0% | 7 | 15.6% | 9 | 10.6% |  |
|  | Not specified | 8 | 20.0% | 11 | 24.4% | 19 | 22.4% |  |
|  | Total | 40 | 100.0% | 45 | 100.0% | 85 | 100.0% |  |

**Abbreviation:** n, valid number; X^2^, Pearson’s Chi-square test.

**Suppl. Table S15** Pre-existing and disease-related patterns of patients without and with psycho-oncological care in cohort III (n = 85).

|  | | Psycho-oncological care | | | | | |  |
| --- | --- | --- | --- | --- | --- | --- | --- | --- |
|  |  | No | | Yes | | Total | | X^2^ |
|  |  | n | (%) | n | (%) | n | (%) | *p* |
| Symptomatic epilepsy  (last visit) | No | 12 | 30.0% | 14 | 31.1% | 26 | 30.6% | 0.912 |
|  | Yes | 28 | 70.0% | 31 | 68.9% | 59 | 69.4% |  |
|  | Total | 40 | 100.0% | 45 | 100.0% | 85 | 100.0% |  |
| Depression  (last visit) | No | 35 | 87.5% | 35 | 77.8% | 70 | 82.4% | 0.241 |
|  | Yes | 5 | 12.5% | 10 | 22.2% | 15 | 17.6% |  |
|  | Total | 40 | 100.0% | 45 | 100.0% | 85 | 100.0% |  |
| Palliative care | No | 16 | 40.0% | 16 | 35.6% | 32 | 37.6% | 0.889 |
|  | Yes | 21 | 52.5% | 26 | 57.8% | 47 | 55.3% |  |
|  | No data available | 3 | 7.5% | 3 | 6.7% | 6 | 7.1% |  |
|  | Total | 40 | 100.0% | 45 | 100.0% | 85 | 100.0% |  |
| In need of POC^*^  (last visit) | No (< 4) | 13 | 56.5% | 13 | 41.9% | 26 | 48.1% | 0.289 |
|  | Yes (≥ 4) | 10 | 43.5% | 18 | 58.1% | 28 | 51.9% |  |
|  | Total | 23 | 100.0% | 31 | 100.0% | 54 | 100.0% |  |
| Surgery at tumor progression | 1 | 10 | 76.9% | 12 | 70.6% | 22 | 73.3% | 0.104 |
|  | 2 | 3 | 23.1% | 5 | 29.4% | 8 | 26.7% |  |
|  | Total | 13 | 100.0% | 17 | 100.0% | 30 | 100.0% |  |
| Radiotherapy at tumor progression | 1 | 11 | 100.0% | 15 | 93.8% | 26 | 96.3% | 0.052 |
|  | 2 | 0 | 0.0% | 1 | 6.3% | 1 | 3.7% |  |
|  | Total | 11 | 100.0% | 16 | 100.0% | 27 | 100.0% |  |
| Chemotherapy at tumor progression | 1 | 7 | 35.0% | 14 | 48.3% | 21 | 42.9% | 0.356 |
|  | 2 | 13 | 65.0% | 15 | 51.7% | 28 | 57.1% |  |
|  | Total | 20 | 100.0% | 29 | 100.0% | 49 | 100.0% |  |

**Abbreviation:** POC, Psycho-oncological care; n, valid number; X^2^, Pearson’s Chi-square test.

^*^Measured with Hornheider Screening Instrument.

**Suppl. Table S16** Results from univariable and multiple linear regression analysis for the correlation of psycho-oncological care and the total mean number of emergency presentations and their reasons in the last three months before death in cohort III (n = 85).

| Total mean number and reasons for emergency presentation | Univariable linear regression analysis | | | | | Multiple linear regression analysis^*^ | | | |
| --- | --- | --- | --- | --- | --- | --- | --- | --- | --- |
|  | *p* | t | B^#^ | Adjusted R Square | *p* | | t | B^#^ | Adjusted R Square |
| Total mean number | 0.564 | -0.579 | -0.063 | -0.008 | 0.267 | | -1.118 | -0.120 | 0.070 |
| Brain edema | 0.023 | 2.321 | 0.247 | 0.050 | 0.007 | | 2.818 | 0.324 | 0.206 |
| Seizure | 0.267 | -1.118 | -0.122 | 0.003 | 0.052 | | -1.980 | -0.240 | 0.087 |
| Pain | 0.389 | 0.866 | 0.095 | -0.003 | 0.581 | | 0.554 | 0.060 | 0.039 |
| Infection | 0.522 | 0.643 | 0.070 | -0.007 | 0.143 | | 1.489 | 0.202 | 0.111 |
| Side effects of therapy | 0.976 | 0.031 | 0.003 | -0.012 | 0.696 | | 0.394 | 0.054 | 0.035 |
| Symptoms due to progression | 0.458 | -0.745 | -0.082 | -0.005 | 0.129 | | -1.545 | -0.199 | 0.156 |
| Others | 0.244 | -1.173 | -0.128 | 0.004 | 0.220 | | -1.242 | -0.157 | 0.168 |

^*^Selected clinical aspects (sex, age at diagnosis in years, histological diagnosis, KPS six months before death, the total number of surgeries, radiotherapies, chemotherapies for progression therapy, symptomatic epilepsy, depression at the last visit, palliative care, need for psycho-oncological care, measured with Hornheider Screening Instrument, at the last visit) were tested for significance at level 0.2 and, if they met the criterion, were included in the multiple linear regression analysis for adjustment. ^#^B: Regression coefficient: positive coefficient represents a greater mean number with psycho-oncological care than without psycho-oncological care.
